# Draft genome assembly of the Aral barbell *Luciobarbus brachycephalus* using PacBio sequencing

**DOI:** 10.1093/gbe/evab131

**Published:** 2021-07-13

**Authors:** Longwu Geng, Ming Zou, Haifeng Jiang, Minghui Meng, Wei Xu

**Affiliations:** 1 Heilongjiang River Fisheries Research Institute, Chinese Academy of Fishery Sciences, Key Open Laboratory of Cold Water Fish Germplasm Resources and Breeding of Heilongjiang Province, Harbin, China; 2 Institute of Zoology, Chinese Academy of Sciences, Beijing, China; 3 College of Animal Science and Technology, Northwest A&F University, Xinong Road 22nd, Yangling, China; 4 Diggs (Wuhan) Biotechnology Co., Ltd

**Keywords:** *Luciobarbus brachycephalus*, PacBio sequencing, *de novo* assembly, genome annotation, phylogeny

## Abstract

The endangered Aral barbell *Luciobarbus brachycephalus* is endemic to the water systems of the Caspian Sea and Aral Sea. Given the scarcity of genetic data for the species, we present a draft assembly based on PacBio long read sequencing technology. Approximate 299.4 Gb of long reads representing 166X of the estimated genome size were generated, and the final assembly was composed of 653 contigs totaling approximately 1,698.3 Mb, with a contig N50 length of 4.5 Mb. A total of 807.6 Mb represented approximately 47.6% of the assembly and were identified as repeats. Fifty-four thousand and six hundred possible protein genes were predicted, among which 50,727, representing approximately 92.9%, could be annotated by at least one database. Evolutionary analysis showed that *L*. *brachycephalus* and *Labeo rohita* diverged by approximately 42.6 Mya, and the obvious expansion of gene families residing in the *L*. *brachycephalus* genome may be attributed to the specific whole genome duplication of the species. The first genome assembly of *L*. *brachycephalus* can not only provide a foundation for genetic conservation and molecular breeding of this species but also contribute to comparative analyses of genome biology and evolution within Cyprinidae.

### Significance

Aral barbell *Luciobarbus brachycephalus* is an endangered fish species endemic to the water systems of the Caspian Sea and Aral Sea. At present, genetic and genomic information for the species and the genus *Brachycephalus* is limited. In this study, we obtained a draft genome assembly of *L. brachycephalus*, which will contribute to research on the genomics, evolution, and genetic breeding of this species and the largest and most diverse fish family, Cyprinidae.

### Introduction

Aral barbell, *Luciobarbus brachycephalus,* is a valuable fish species native to the Aral basin, Chu drainage and southern and western Caspian Sea (Coad 1998). It has been speculated that the population of *L. brachycephalus* has declined by at least 30% in the past 30 years and continues to decline due to the shrinking (increased salinity) of the Aral Sea and damming of its tributaries. Aral barbel is currently listed on the IUCN Red List as a vulnerable (VN) species (Esmaeili, et al. 2017). However, a lack of information on genetic and genomic information for this endangered species hinders efforts to protect, restore and effectively manage the Aral barbell population.

The Aral barbell belongs to the Barbinae subfamily, family Cyprinidae (Cypriniformes), in which polyploidization evolved independently on multiple occasions (Xu, et al. 2019). Cyprinids contain many autoallopolyploid fish species, resulting in complicated evolutionary histories and phylogenetic relationships. The chromosome number of *L. brachycephalus* is 100 (Geng, et al. 2013), twice that in the common ancestor of Barbinae. It appears that *L. brachycephalus* has experienced a polyploidization event beyond teleost-specific whole genome duplication (WGD). Polyploidization plays a significant role in speciation and adaptive evolution since it produces redundant genes, which can serve as an important genetic material basis for complex phenotypes (Xu, et al. 2019). Unlike plant lineages where polyploidization occurs frequently (Mayrose and Lysak 2020; Wu, et al. 2021), vertebrate polyploidization is relatively rare, and is mainly concentrated in some amphibians and fish (Chen, et al. 2020). The polyploid history of *L. brachycephalus* provides an excellent model to explore the evolutionary patterns and histories of polyploidization in vertebrates.

*L. brachycephalus* has high potential for aquaculture exploitation in saline alkali water due to its strong saline-alkali tolerance (salinity < 10 g/L, alkalinity < 30 mmol/L) (Geng, et al. 2016). Therefore, *L. brachycephalus* was introduced into China from Uzbekistan as the name large-scale barbell in 2003 (Jiang, et al. 2019). Since then, a great deal of studies on artificial breeding, biological performance and larval rearing have been conducted (Li, et al. 2019; Longwu, et al. 2010). At present, *L. brachycephalus* is becoming more economically important owing to its taste, fast growth and high commercial value, and it has been cultured in more than 20 provinces in China with an annual production of up to 20,000 - 40,000 tons. However, the genetic resources of *L. brachycephalus* are relatively limited, and the genetic diversity is generally low due to founder effects and the inbreeding of small populations. Therefore, it is urgent to develop genomic resources to accelerate the genome-assisted breeding of *L. brachycephalus*. However, to date, only the mitochondrial genome and microsatellite DNA have been reported for the Aral barbell (Jiang, et al. 2019; Longwu, et al. 2012). Here, we report a draft genome assembly of *L. brachycephalus*, which will provide valuable genomic resources for studies on conservation and breeding as well as polyploid origin, speciation and adaptation in polyploid Cyprinidae.

### Results and Discussion

#### Genome Sequencing and Assembly

Based on the genomic data generated in the present study, the whole genome of *L. brachycephalus* was assembled. More than 675,436,128 paired-end reads with a length of 150 bp totaling more than 101.3 Gb were generated for the survey analysis (supplementary table S1). The estimated genome size for the species was approximately 1,806.6 Mb (supplementary table S2). The features of the species were similar to those of polyploid-related species such as *C. carpio* and *Schizothorax o'connori* (Xiao, et al. 2020; Xu, et al. 2014). Thus, the genome of *L. brachycephalus* may be tetraploid. The *de novo* assembly following rounds of polishing generated an assembly composed of 653 contigs totaling 1698.3 Mb, with a contig N50 length of 4.5 Mb (supplementary table S3). BUSCO assessment showed that approximately 96.0% of the BUSCO genes were recovered completely by assembly, of which 37.8% were single copy and 58.2% were duplicated (supplementary table S4). The assembly may partially recover another 1.6% of the BUSCO genes (supplementary table S4). In this study, approximately 40 M paired-end reads totaling nearly 7 Gb were generated each for the brain, heart, kidney, liver, and ovary (supplementary table S1). The mapping rate for the RNA-Seq data to the new assembly reached more than 80% for each of the tissues. Thus, the assembly of the *L. brachycephalus* genome may be of high quality.

#### Genome Annotation

A number of repeat elements and protein-coding genes residing in the new assembly were identified in the study. All the identified repetitive elements occupied 47.55% of the genome (supplementary tables S5 and S6), which was higher than that of *C. carpio* but lower than that of *S. o'connori* (Xiao, et al. 2020; Xu, et al. 2014). Briefly, RepeatModeler + RepeatMasker identified that approximately 33.8% of the genome was repeats. Then, on the basis of consensus sequences deposited in Repbase, RepeatMasker identified approximately 28.5% of the genome as repeats (supplementary table S6). RepeatProteinMask and LTR_retriever identified approximately 11.4% and 6.1% of the genomes as possible TE proteins and LTRs, respectively (supplementary table S6). Moreover, more than 1.3 M SSRs were identified from the assembly of the *L. brachycephalus* genome.

The process of gene-model annotation identified a total of 54,600 protein-coding genes residing in the *L. brachycephalus* genome with a mean transcript length of 15,737.16 bp. The gene number of *L. brachycephalus* is much greater than that of diploid relatives such as *D. rerio* and C. *idellus* (Howe, et al. 2013; Wang, et al. 2015) but is similar to that of tetraploids *C. carpio* and *S. o'connori* (Xiao, et al. 2020; Xu, et al. 2014). A total of 50,727 genes accounting for approximately 92.9% of the total genes were annotated against at least one database (supplementary table S7). Among these databases, NR annotated 50,477 genes, accounting for 92.5% of the total genes, followed by SwissProt, which annotated 81.4% (supplementary table S7).

**Comparative Genome Analysis**

The evolutionary dynamics related to *L. brachycephalus* were deduced by comparison to the relative species. Seven hundred thirty-two one-to-one orthologs were identified among these species (fig. 1), and the concatenated supermatrix of the alignments of these genes suggested that *L. brachycephalus* and *L. rohita* formed sister groups with high confidence (fig. 1). Divergence time estimation suggested that *L. brachycephalus* and *L. rohita* diverged by approximately 42.6 Mya. Approximately 6,306 and 338 gene families were probably subject to expansions and contractions in the species, respectively (fig. 1). The obvious expansion of gene families within the *L. brachycephalus* genome may coincide with its whole genome duplication (Voldoire, et al. 2017).

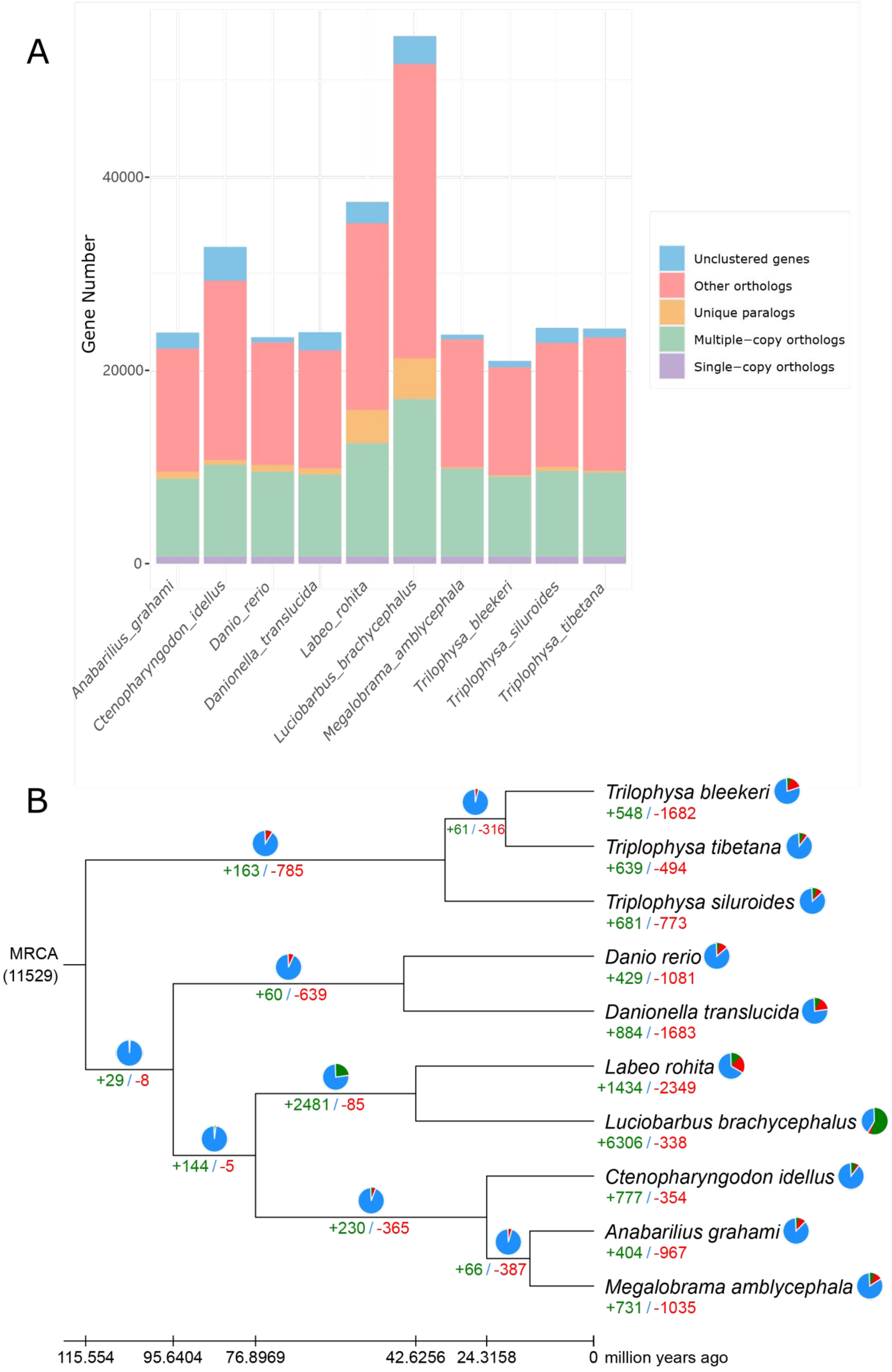

A
Gene Number
40000
20000
0
Unclustered genes
Other orthologs
Unique paralogs
Multiple−copy orthologs
Single−copy orthologs
Anabarilius_grahami
Ctenopharyngodon_idellus
Danio_rerio
Danionella_translucida
Labeo_rohita
Luciobarbus_brachycephalus
Megalobrama_amblycephala
Trilophysa_bleekeri
Triplophysa_siluroides
Triplophysa_tibetana
B
MRCA
(11529)
Trilophysa bleekeri
+548 / -1682
Triplophysa tibetana
+639 / -494
Triplophysa siluroides
+681 / -773
Danio rerio
+429 / -1081
Danionella translucida
+884 / -1683
Labeo rohita
+1434 / -2349
Luciobarbus brachycephalus
+6306 / -338
Ctenopharyngodon idellus
+777 / -354
Anabarilius grahami
+404 / -967
Megalobrama amblycephala
+731 / -1035
+61 / -316
+163 / -785
+60 / -639
+29 / -8
+2481 / -85
+144 / -5
+230 / -365
+66 / -387
115.554
95.6404
76.8969
42.6256
24.3158
0 million years ago

**FIG. 1.**—Comparison between *L. brachycephalus* and relative species. (A) Comparison of copy numbers in gene clusters reside in the genomes of *L. brachycephalus* and the other 9 relative fish species. Single-copy orthologs denote the family have and only have one gene for each species, and multi-copy orthologs denote the family clustered more than one gene for each species. Other orthologs denote the family can have any number of genes for each species except the single-copy and multi-copy orthologs. Unique paralogs denote species-specific gene families, and Unclustered genes denote species-specific genes cannot cluster with any other genes. (B) Phylogenetic relationships between *L. brachycephalus* and relatives recovered based on the maximum likelihood method. All nodes are fully supported by the bootstrap resampling test. The numbers and sectors marked with green, red, and blue denote gene families subject to expansion, contraction, and stability for each species, respectively.

### Conclusions

In the present study, we sequenced and *de novo* assembled the whole genome of tetraploid *L. brachycephalus* based on PacBio sequencing technology. The estimated genome size of the species is approximately 1,806.6 Mb, and the assembly recovered more than 94.0% of the estimated size. Approximately 47.55% of the assembly may be composed of repeat elements, and a total of 54,600 protein genes were identified. The first genome sequences for the species should be a valuable resource for studies on the genomics, evolution, and ecology of polyploid fish in the family Cyprinidae.

### Materials and Methods

#### Sample Collection and Sequencing

An individual sample of *L. brachycephalus* was obtained from a breeding farm at Hulan Experimental Station, Heilongjiang River Fisheries Research Institute (Harbin, P. R. China), for genome sequencing. After the injection of tricaine methanesulfonate (MS-222), it was dissected, and a number of tissues, including white muscle, brain, heart, kidney, liver, and ovary, were sampled and immediately frozen in liquid nitrogen. For genome sequencing, genomic DNA from white muscle was extracted using a standard phenol/chloroform method. The integrity of the extracted DNA was assessed by 0.75% agarose gel electrophoresis, and the concentration was quantified by a Qubit 4 Fluorometer (Thermo Fisher Scientific, Inc., USA). Ten micrograms of DNA was then used to construct the library for PacBio SMRT Sequencing using the SMRTbell Express Template Prep Kit (PacBio, Menlo Park, CA, USA), and the library was sequenced using the PacBio Sequel II System with CLR mode. To estimate the profiles, including genome size, heterozygosity, and repeat contents of the genome, a paired-end library with a 400 bp insert size was constructed using genomic DNA with an Illumina TruSeq DNA Nano Preparation Kit (Illumina, San Diego, CA, USA) and sequenced using the Illumina HiSeq Xten platform (Illumina). To assist the annotation of gene models, total RNA from all the sampled tissues, that is, brain, heart, kidney, liver, and ovary, was extracted using TRIzol reagent (Invitrogen, Carlsbad, CA) following the manufacturer's protocol, and libraries with 400 bp insert sizes were prepared and sequenced based on the Illumina HiSeq Xten platform. All experiments involving the handling and treatment of fish in this study were approved by the Animal Care and Use Committee of the HRFRI of the Chinese Academy of Fishery Sciences.

**Genome Assembly and Assessment**

The Illumina short reads generated for the survey were quality controlled using fastp (Chen, et al. 2018) and were used to estimate the genome profiles using GenomeScope2 (Ranallo-Benavidez, et al. 2020) based on the 17-mer counts generated by Jellyfish2.0 (Marcais and Kingsford 2011). Based on the estimated genome size, the genomic long reads were used to *de novo* assemble the genome using FALCON 0.3.0 (Chin, et al. 2016) with the following parameters: "length_cutoff = 21000, length_cutoff_pr = 20000, pa_HPCdaligner_option = -v -B4 -t16 -e.70 -l1000 -s1000 -T12 -M50, overlap_filtering_setting = --bestn 10 --n_core 16 --min_cov 3 --max_diff 100 --max_cov 100". To improve the assembly, all the long reads were mapped back to the new assembly using blasr v5.3.1 with default settings (Chaisson and Tesler 2012) and were used to polish the genome using gcpp2 (https://github.com/PacificBiosciences/gcpp). The improved assembly was also polished using pilon v1.8 (Walker, et al. 2014) based on the alignments of genomic short reads mapped back to the assembly using BWA-MEM with default settings (Li 2013). The process was repeated 3 times iteratively to try to adjust the assembly to increase its accuracy. Evolutionarily informed expectations of the gene content of near-universal single-copy orthologs within Actinopterygii were estimated using Benchmarking Universal Single-Copy Orthologs (BUSCO 3) software to estimate the completeness of the new assembly (Simão, et al. 2015). To estimate the accuracy of the new assembly, all the cleaned short reads from each of the RNA-Seq libraries were mapped back to the genome using hisat2 with default settings (Kim, et al. 2015), and the mapping rate was estimated.

### The Annotation of Repetitive Elements

Two strategies were recruited to identify repeats residing in the genome. The first was *de novo* identification based on structural characters, and the second was based on homology to known sequences. On the basis of their structural characteristics, tandem repeats were detected by tandem repeats finder TRF v4.07b (Benson 1999), and simple sequence repeats (SSRs) residing in the genome were identified using MISA (Beier, et al. 2017) with default settings. Long terminal repeats (LTRs) were identified using LTR_retriever (Ou and Jiang 2018) on the basis of the results of LTRharvest (Ellinghaus, et al. 2008) and LTR_Finder (Ou and Jiang 2019) with the suggested parameters described in the manual. The *de novo* identification of other repeats was implemented using RepeatModeler followed by genome-scale detection using RepeatMasker v4.0.6 (Tarailo-Graovac and Chen 2009) based on homology to the consensus sequences. The consensus sequences deposited in Repbase were also used for the genome-scale detection of repeat regions residing in the *L. brachycephalus* genome using RepeatMasker v4.0.6. Possible TE proteins were detected by RepeatProteinMask. All aforementioned results were combined and merged to generate a nonredundant list of repeat elements residing in the genome.

### Gene Prediction and Functional Annotation

Three strategies are typically used to identify gene models residing in eukaryotic genomes: ab initio, homology-based, and transcriptome-based predictions. The ab initio prediction of genes was performed using Augustus (Stanke, et al. 2006), GlimmerHMM (Majoros, et al. 2004) and Geneid (Blanco, et al. 2007). The model parameters for these tools were trained based on the genes predicted during BUSCO v.3.0.1 (Simão, et al. 2015) estimation of the assembly completeness. For the prediction based on homology, proteomes downloaded from public databases for 11 relatives, including *Hypophthalmichthys molitrix*, *Danio rerio*, *Megalobrama amblycephala*, *Trilophysa bleekeri*, *Triplophysa siluroides*, and *Triplophysa tibetana,* were used to scan the genome of *L. brachycephalus* using tblastn (Camacho, et al. 2009) with an E-value ≤ 1e-05, and GeneWise v.2.4.0 (Birney, et al. 2004) was used to align the proteins to the homologous genome sequences for accurate spliced alignments. The generated RNA-seq data were also used to obtain direct evidence of the protein coding sequences following the PASA pipeline (Haas, et al. 2008). The results from the above gene prediction methods were merged by EVM (Haas, et al. 2008) with different weights. After that, all the genes with transposable elements were removed by TransposonPSI to obtain the final gene set (http://transposonpsi.sourceforge.net). The gene set was annotated by homology scanning to proteins deposited in SwissProt, the nonredundant dataset (NR), and KEGG using Blastp (Camacho, et al. 2009) with an E-value of 1e-05. Gene Ontology (GO) and protein domain annotations were implemented using InterProScan (Quevillon, et al. 2005) with default settings.

**Comparative analyses with relative species**

By comparing the predicted protein sequences of *L. brachycephalus* with those of the related species, including *Anabarilius grahami*, *Ctenopharyngodon idellus*, *Danionella translucida*, *D. rerio*, *Labeo rohita*, *M. amblycephala*, *T. bleekeri*, *T. siluroides*, and *T. tibetana*, their phylogenetic relationships, divergence times, and the expansions and contractions of gene families were deduced. First, OrhoMCL v2.0 was used to cluster gene families among these species with default settings (Li, et al. 2003). One-to-one gene families among these species were then selected and aligned using MAFFT V7 (Katoh, et al. 2002). After that, all the alignments were concatenated to form a supermatrix and were used to deduce their phylogenetic relationships using RAxML7 (Stamatakis 2015). One hundred rapid bootstraps were also implemented using RAxML7 to estimate the robustness of the phylogeny (Stamatakis, et al. 2008). Based on the phylogeny and the 4-fold degenerate sites extracted from the concatenated supermatrix, their divergence times were estimated using MCMCTREE included in the PAML4 software package (Yang 2007). Based on the estimated divergence times and the contents for each gene family clustered using OrhoMCL, the possible gene families subject to expansions and contractions for each species were deduced using CAFÉ2 (De Bie, et al. 2006).

### Acknowledgments

We appreciate Pan Xu, general manager of Diggs (Wuhan) Biotechnology Co., Ltd., for his helpful suggestions on genome assembly. This study was supported by the National Key R & D Program of China (2019YFD0900405), the Heilongjiang province natural science foundation of China (LH2019C088), the

Central Public-interest Scientific Institution Basal Research Fund. HRFRI [NO. HSY202013PT, HSY201809M, HSY201803M], the Central Public-interest Scientific Institution Basal Research Fund, CAFS under Grant [2020TD56].

### Author Contributions

L.G., H.J. and W.X. conceived the study and collected the samples for sequencing. M.M. performed the genomics analysis. L.G., H.J. and M.Z. wroted and revised the manuscript. All authors approved the final submission.

### Data Availability

All raw sequencing data, genome assembly and annotations for this article have been deposited at Genome Sequence Archive (GSA, https://bigd.big.ac.cn/gsa/) under accession number PRJCA004709.

## References

Beier S, Thiel T, Münch T, Scholz U, Mascher M. 2017. MISA-web: a web server for microsatellite prediction. Bioinformatics 33: 2583-2585.

Benson G. 1999. Tandem repeats finder: a program to analyze DNA sequences. Nucleic Acids Research 27: 573-580. doi: 10.1093/nar/27.2.573.

Birney E, Clamp M, Durbin R. 2004. GeneWise and genomewise. Genome Research 14: 988-995. doi: 10.1101/gr.1865504.

Blanco E, Parra G, Guigó R. 2007. Using geneid to Identify genes. Current Protocols in Bioinformatics 18: 4.3.1-4.3.28. doi: 10.1002/0471250953.bi0403s18.

Camacho C, et al. 2009. BLAST+: architecture and applications. BMC Bioinformatics 10: 421. doi: 10.1186/1471-2105-10-421.

Chaisson MJ, Tesler G. 2012. Mapping single molecule sequencing reads using basic local alignment with successive refinement (BLASR): application and theory. BMC Bioinformatics 13: 238. doi: 10.1186/1471-2105-13-238.

Chen S, Zhou Y, Chen Y, Gu J. 2018. fastp: an ultra-fast all-in-one FASTQ preprocessor. Bioinformatics 34: i884-i890. doi: 10.1093/bioinformatics/bty560.

Chin CS, et al. 2016. Phased diploid genome assembly with single-molecule real-time sequencing. Nat Methods 13: 1050-1054. doi: 10.1038/nmeth.4035.

Coad BW. 1998. Systematic biodiversity in the freshwater fishes of Iran. Canadian Journal of Physiology and Pharmacology 65: 101-108.

De Bie T, Cristianini N, Demuth JP, Hahn MW. 2006. CAFE: a computational tool for the study of gene family evolution. Bioinformatics 22: 1269-1271. doi: 10.1093/bioinformatics/btl097.

Ellinghaus D, Kurtz S, Willhoeft U. 2008. LTRharvest, an efficient and flexible software for de novo detection of LTR retrotransposons. BMC Bioinformatics 9: 18. doi: 10.1186/1471-2105-9-18.

Esmaeili HR, Mehraban H, Abbasi K, Keivany Y, Coad BW. 2017. Review and updated checklist of freshwater fishes of Iran: taxonomy, distribution and conservation status. Iranian Journal of Ichthyology 4: 1-114.

Geng L, Tong G, Jiang H, Wei X. 2016. Effect of salinity and alkalinity on *Luciobarbus capito* gill $Na^+/K^+$-ATPase enzyme activity, plasma ion concentration, and osmotic pressure. BioMed Research International 2016: 1-7.

Geng LW, Xu W, Jiang HF, Tong GX. 2013. Karyotype analysis of *Barbus capito* (Güldentstädt, 1773) using curve measurement software. Journal of Applied Ichthyology 29: 922-924.

Haas BJ, et al. 2008. Automated eukaryotic gene structure annotation using EVidenceModeler and the program to assemble spliced alignments. Genome Biology 9: R7. doi: 10.1186/gb-2008-9-1-r7.

Howe K, et al. 2013. The zebrafish reference genome sequence and its relationship to the human genome. Nature 496: 498-503. doi: 10.1038/nature12111.

Jiang H, Geng L, Yang J, Tong G, Xu W. 2019. The complete mitochondrial genome of the aral barbel *Luciobarbus brachycephalus* (Cypriniformes: Cyprinidae: Barbinae). Mitochondrial DNA Part B 4: 3685-3686.

Katoh K, Misawa K, Kuma Ki, Miyata T. 2002. MAFFT: a novel method for rapid multiple sequence alignment based on fast Fourier transform. Nucleic Acids Research 30: 3059-3066. doi: 10.1093/nar/gkf436.

Kim D, Langmead B, Salzberg SL. 2015. HISAT: a fast spliced aligner with low memory requirements.

Nat Methods 12: 357-360. doi: 10.1038/nmeth.3317.

Li A, et al. 2019. Morphological characteristics and parameters measurement of *Barbus capito*. Chinese Agricultural Science Bulletin. p. 144-152.

Li H. 2013. Aligning sequence reads, clone sequences and assembly contigs with BWA-MEM. arXiv 1303.3997.

Li L, Stoeckert CJ, Roos DS. 2003. OrthoMCL: Identification of ortholog groups for eukaryotic genomes. Genome Research 13: 2178-2189. doi: 10.1101/gr.1224503.

Longwu G, Cuiyun L, Guangxiang T, Chao L, Wei X. 2012. Development and characterization of twenty microsatellite markers for the endangered fish Luciobarbus capito. Conservation Genetics Resources 4: 865-867.

Longwu G, et al. 2010. Technique of artificial reproduction of *Barbus capito*. Journal of Jilin Agricultural University. p. 218-220.

Majoros WH, Pertea M, Salzberg SL. 2004. TigrScan and GlimmerHMM: two open source ab initio eukaryotic gene-finders. Bioinformatics 20: 2878-2879. doi: 10.1093/bioinformatics/bth315.

Marcais G, Kingsford C. 2011. A fast, lock-free approach for efficient parallel counting of occurrences of k-mers. Bioinformatics 27: 764-770. doi: 10.1093/bioinformatics/btr011.

Ou S, Jiang N. 2019. LTR_FINDER_parallel: parallelization of LTR_FINDER enabling rapid identification of long terminal repeat retrotransposons. Mob DNA 10: 48. doi: 10.1186/s13100-019-0193-0.

Ou S, Jiang N. 2018. LTR_retriever: a Highly accurate and sensitive program for identification of long terminal repeat retrotransposons. Plant Physiol 176: 1410-1422. doi: 10.1104/pp.17.01310.

Quevillon E, et al. 2005. InterProScan: protein domains identifier. Nucleic Acids Research 33: W116-W120.

Ranallo-Benavidez TR, Jaron KS, Schatz MC. 2020. GenomeScope 2.0 and Smudgeplot for reference-free profiling of polyploid genomes. Nat Commun 11: 1432. doi: 10.1038/s41467-020-14998-3.

Simão FA, Waterhouse RM, Ioannidis P, Kriventseva EV, Zdobnov EM. 2015. BUSCO: assessing genome assembly and annotation completeness with single-copy orthologs. Bioinformatics 31: 3210-3212. doi: 10.1093/bioinformatics/btv351.

Stamatakis A. 2015. Using RAxML to infer phylogenies. Current Protocols in Bioinformatics 51: 6.14.11-16.14.14. doi: 10.1002/0471250953.bi0614s51.

Stamatakis A, Hoover P, Rougemont J. 2008. A rapid bootstrap algorithm for the RAxML Web servers. Syst Biol 57: 758-771. doi: 10.1080/10635150802429642.

Stanke M, et al. 2006. AUGUSTUS: ab initio prediction of alternative transcripts. Nucleic Acids Research 34: W435-W439. doi: 10.1093/nar/gkl200.

Tarailo-Graovac M, Chen N. 2009. Using repeatMasker to identify repetitive elements in genomic sequences. Current Protocols in Bioinformatics 25: 4.10.11-14.10.14. doi: 10.1002/0471250953.bi0410s25.

Voldoire E, Brunet F, Naville M, Volff J-N, Galiana D. 2017. Expansion by whole genome duplication and evolution of the sox gene family in teleost fish. PloS one 12: e0180936-e0180936. doi: 10.1371/journal.pone.0180936.

Walker BJ, et al. 2014. Pilon: an integrated tool for comprehensive microbial variant detection and genome assembly improvement. PLoS One 9: e112963. doi: 10.1371/journal.pone.0112963.

Wang Y, et al. 2015. The draft genome of the grass carp (Ctenopharyngodon idellus) provides insights into its evolution and vegetarian adaptation. Nature Genetics 47: 625-631. doi: 10.1038/ng.3280.

Xiao S, et al. 2020. Genome of tetraploid fish schizothorax o'connori provides insights into early re-diploidization and high-altitude adaptation. iScience 23: 101497. doi: 10.1016/j.isci.2020.101497.

Xu P, et al. 2019. The allotetraploid origin and asymmetrical genome evolution of the common carp Cyprinus carpio. Nat Commun 10: 4625. doi: 10.1038/s41467-019-12644-1.

Xu P, et al. 2014. Genome sequence and genetic diversity of the common carp, Cyprinus carpio. Nature Genetics 46: 1212-1219. doi: 10.1038/ng.3098.

Yang Z 2007. PAML 4: phylogenetic analysis by maximum likelihood. Molecular Biology and Evolution 24: 1586-1591. doi: 10.1093/molbev/msm088.

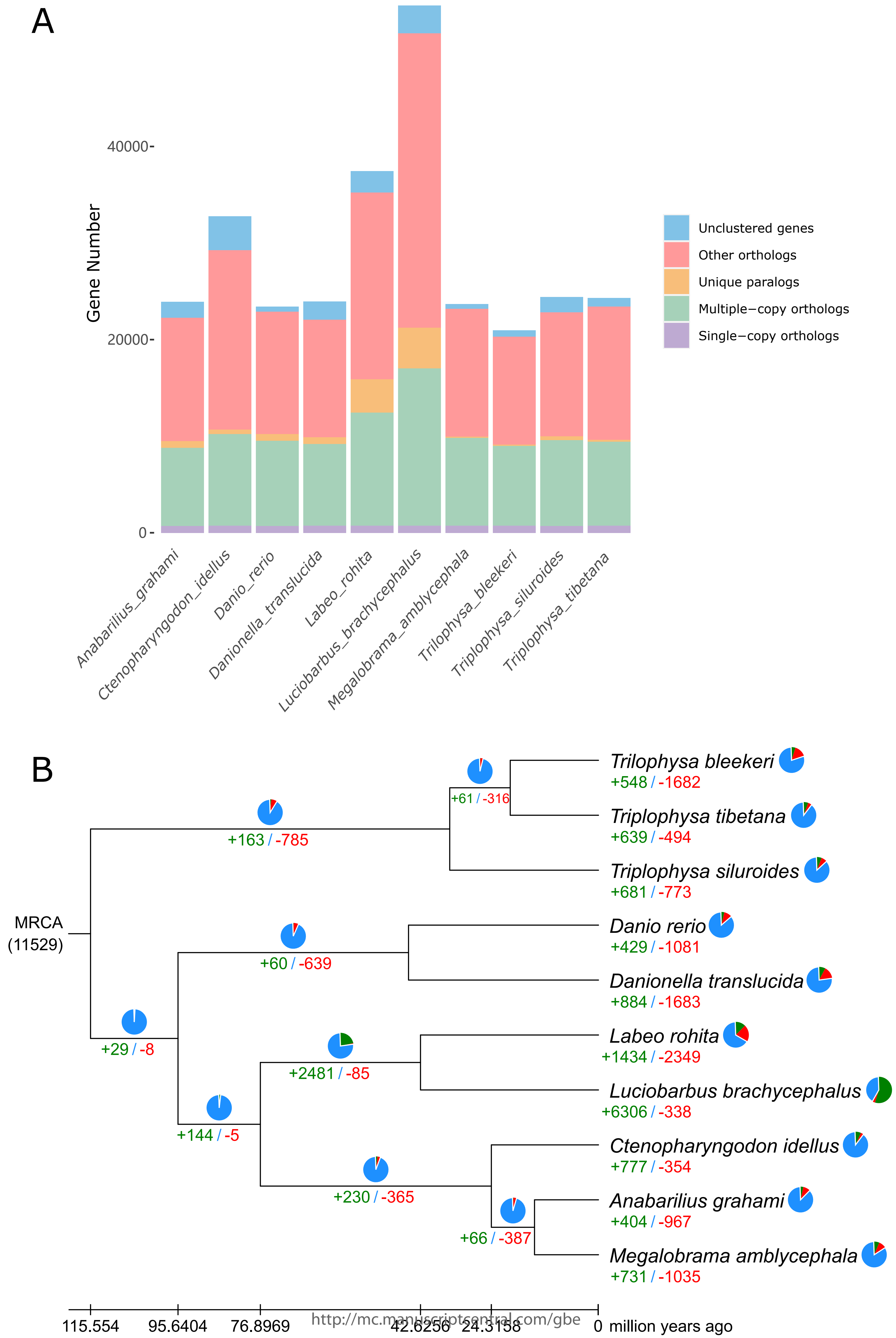

A
Gene Number
40000
20000
0
Unclustered genes
Other orthologs
Unique paralogs
Multiple−copy orthologs
Single−copy orthologs
Anabarilius_grahami
Ctenopharyngodon_idellus
Danio_rerio
Danionella_translucida
Labeo_rohita
Luciobarbus_brachycephalus
Megalobrama_amblycephala
Trilophysa_bleekeri
Triplophysa_siluroides
Triplophysa_tibetana
B
MRCA
(11529)
+29 / -8
+163 / -785
+61 / -316
+60 / -639
+144 / -5
+2481 / -85
+230 / -365
+66 / -387
Trilophysa bleekeri
+548 / -1682
Triplophysa tibetana
+639 / -494
Triplophysa siluroides
+681 / -773
Danio rerio
+429 / -1081
Danionella translucida
+884 / -1683
Labeo rohita
+1434 / -2349
Luciobarbus brachycephalus
+6306 / -338
Ctenopharyngodon idellus
+777 / -354
Anabarilius grahami
+404 / -967
Megalobrama amblycephala
+731 / -1035
115.554
95.6404
76.8969
42.6256
24.3158
0 million years ago

## Supplementary Material

evab131_Supplementary_Data

