# Supplementary material for: Draft genome assembly of the Aral barbell Luciobarbus brachycephalus using PacBio sequencing
Source: Genome Biol Evol. 2021 Jul 13;13(7):evab131. doi: 10.1093/gbe/evab131 (PMC8489429; doi:10.1093/gbe/evab131)
Supplement: evab131_Supplementary_Data [file evab131_Supplementary_Data.zip › suppl_data/revised supplementary tables(S1-S7)(MS GBE-210442).docx]

**Supplementary Table S1** Raw data generated for *L. brachycephalus* in the present study.

| Sequencing type | application | tissue | platform | Number of raw data sequences | Size of raw data (Gb) |
| --- | --- | --- | --- | --- | --- |
| Genome-Seq | Survey | White muscle | Illumina HiSeq Xten | 675,436,128 | 101.3 |
|  | *De novo* |  | PacBio Sequel II | 14,651,985 | 299.4 |
| RNA-Seq | Assist annotation | brain | Illumina HiSeq Xten | 42,875,144 | 6.4 |
|  |  | heart |  | 44,267,786 | 6.6 |
|  |  | kidney |  | 43,809,308 | 6.6 |
|  |  | liver |  | 39,630,746 | 5.9 |
|  |  | ovary |  | 44,879,810 | 6.7 |

**Supplementary Table S2** Estimation of genome size based on 17-mer statistics.

| Kmer | Depth | N-kmer | Genome size (M) | Heterozygous rate (%) |
| --- | --- | --- | --- | --- |
| 17 | 20 | 36,130,924,647 | 1806.55 | 4.30 |

**Supplementary Table S3** Summary of the *L. brachycephalus* genome assembly.

“Total” denotes all contigs of the entire genome assembly, “Max” denotes the contig with the maximum length, “Number>=5000” denotes all the contigs with length more than 5000bp.

| StatType |  | Length(bp) | number |
| --- | --- | --- | --- |
| Total |  | 1,698,278,157 | 653 |
| Max |  | 24,692,264 | 1 |
| Number>=5000 |  | 1,698,278,157 | 653 |
| N50 |  | 4,467,962 | 112 |
| N60 |  | 3,588,938 | 155 |
| N70 |  | 2,706,937 | 209 |
| N80 |  | 2,129,766 | 280 |
| N90 |  | 1,421,911 | 376 |

**Supplementary Table S4** BUSCO assessment of the *L. brachycephalus* genome assembly.

| Stats |  | BUSCO notation assessment results |
| --- | --- | --- |
| Total BUSCO groups searched |  | 4,584 |
| Complete and single-copy BUSCOs |  | 37.8% |
| Complete Duplicated BUSCOs |  | 58.2% |
| Fragmented BUSCOs |  | 1.6% |
| Missing BUSCOs |  | 2.4% |

**Supplementary Table S5** The total length and the percentage of the total length account for whole genome of each repeat elements type reside in the *L. brachycephalus* genome**.**

| Type | Length (Mb) | % in genome |
| --- | --- | --- |
| DNA transposons | 397.8 | 23.4 |
| LINE | 112.2 | 6.6 |
| LTR | 178.6 | 10.5 |
| SINE | 11.0 | 0.7 |
| Other | 40.7 | 2.4 |
| Unknown | 67.2 | 4.0 |
| Total | 807.6 | 47.6 |

**Supplementary Table S6** Summary statistics of the repeat elements detected by different strategies and tools.

| Type | Repeat size (bp) | % of genome |
| --- | --- | --- |
| RepeatMasker (RepeatModeler) | 574,082,768 | 33.80 |
| RepeatMasker (Repbase) | 483,421,704 | 28.47 |
| RepeatProteinMask | 193,680,222 | 11.40 |
| LTR_retriever | 103,497,919 | 6.09 |
| Trf | 65,692,898 | 3.87 |
| Total (non-redundant) | 807,590,688 | 47.55 |

**Supplementary Table S7** Summary statistics of the functional annotations for the predicted gene-models of *L. brachycephalus* against each of the Databases.

|  | Database | | Number | Percent (%) |
| --- | --- | --- | --- | --- |
| Annotation | | Swissprot | 44,428 | 81.4 |
|  |  | NR | 50,477 | 92.5 |
|  |  | KEGG | 28,807 | 52.8 |
|  |  | COG | 31,357 | 57.4 |
|  |  | GO | 27,932 | 51.2 |
| Total | | Annotated | 50,727 | 92.9 |
|  |  | Gene | 54,600 | - |
