# Supplementary material for: Draft genome assembly of the Aral barbell Luciobarbus brachycephalus using PacBio sequencing
Source: Genome Biol Evol. 2021 Jul 13;13(7):evab131. doi: 10.1093/gbe/evab131 (PMC8489429; doi:10.1093/gbe/evab131)
Supplement: evab131_Supplementary_Data [file evab131_Supplementary_Data.zip › suppl_data/Supplementary_info_legends_210442.docx]

**Table legends**

**Supplementary Table S1** Raw data generated for *L*. *brachycephalus* in the present study.

**Supplementary Table S2** Estimation of genome size based on 17-mer statistics.

**Supplementary Table S3** Summary of the *L*. *brachycephalus* genome assembly.

“Total” denotes all contigs of the entire genome assembly, “Max” denotes the contig with the maximum length, “Number>=5000” denotes all the contigs with length more than 5000bp.

**Supplementary Table S4** BUSCO assessment of the *L*. *brachycephalus* genome assembly.

**Supplementary Table S5** The total length and the percentage of the total length account for whole genome of each repeat elements type reside in the *L*. *brachycephalus* genome**.**

**Supplementary Table S6** Summary statistics of the repeat elements detected by different strategies and tools.

**Supplementary Table S7** Summary statistics of the functional annotations for the predicted gene-models of *L*. *brachycephalus* against each of the Databases.

**Figure legends**

**FIG. 1.—**Comparison between *L*. *brachycephalus* and relative species. (A) Comparison of copy numbers in gene clusters reside in the genomes of *L*. *brachycephalus* and the other 9 relative fish species. Single-copy orthologs denote the family have and only have one gene for each species, and multi-copy orthologs denote the family clustered more than one gene for each species. Other orthologs denote the family can have any number of genes for each species except the single-copy and multi-copy orthologs. Unique paralogs denote species-specific gene families, and Unclustered genes denote species-specific genes cannot cluster with any other genes. (B) Phylogenetic relationships between *L*. *brachycephalus* and relatives recovered based on the maximum likelihood method. All nodes are fully supported by the bootstrap resampling test. The numbers and sectors marked with green, red, and blue denote gene families subject to expansion, contraction, and stability for each species, respectively.
